# Overexpression of Both *ERG11* and *ABC2* Genes Might Be Responsible for Itraconazole Resistance in Clinical Isolates of *Candida krusei*

**DOI:** 10.1371/journal.pone.0136185

**Published:** 2015-08-26

**Authors:** Xiaoyuan He, Mingfeng Zhao, Jinyan Chen, Rimao Wu, Jianlei Zhang, Rui Cui, Yanyu Jiang, Jie Chen, Xiaoli Cao, Yi Xing, Yuchen Zhang, Juanxia Meng, Qi Deng, Tao Sui

**Affiliations:** 1 Department of Hematology, Tianjin First Central Hospital, The First Central Clinical College of Tianjin Medical University, Tianjin, China; 2 Department of Clinical Laboratory, Tianjin First Central Hospital, Tianjin, China; Instituto de Salud Carlos III, SPAIN

## Abstract

**Objective:**

To study the main molecular mechanisms responsible for itraconazole resistance in clinical isolates of *Candida krusei*.

**Methods:**

The 14α-demethylases encoded by *ERG11* gene in the 16 *C*.*krusei* clinical isolates were amplified by polymerase chain reaction (PCR), and their nucleotide sequences were determined to detect point mutations. Meanwhile, *ERG11* and efflux transporters (*ABC1* and *ABC2*) genes were determined by quantitative real-time reverse transcription polymerase chain reaction (qRT-PCR) for their expression in itraconazole-resistant (R), itraconazole-susceptible dose dependent (SDD) and itraconazole-susceptible (S) *C*.*krusei* at the mRNA level.

**Results:**

We found 7-point mutations in *ERG11* gene of all the *C*.*krusei* clinical isolates, including 6 synonymous mutations and 1 missense mutation (C44T). However, the missense mutation was found in the three groups. The mRNA levels of *ERG11* gene in itraconazole-resistant isolates showed higher expression compared with itraconazole-susceptible dose dependent and itraconazole-susceptible ones (P = 0.015 and P = 0.002 respectively). *ABC2* gene mRNA levels in itraconazole-resistant group was significantly higher than the other two groups, and the levels of their expression in the isolates appeared to increase with the decrease of susceptibility to itraconazole (P = 0.007 in SDD compared with S, P = 0.016 in SDD with R, and P<0.001 in S with R respectively). While *ABC1* gene presented lower expression in itraconazole resistant strains. However, the mRNA levels of *ERG11*, *ABC1* and *ABC2* in a *C*.*krusei* (CK10) resistant to both itraconazole and voriconazole were expressed highest in all the itraconazole-resistant isolates.

**Conclusions:**

There are *ERG11* gene polymorphisms in clinical isolates of *C*.*krusei*. *ERG11* gene mutations may not be involved in the development of itraconazole resistance in *C*.*krusei*. *ERG11* and *ABC2* overexpression might be responsible for the acquired itraconazole resistance of these clinical isolates.

## Introduction

In recent 30 years, the incidence and mortality of fungal infection, especially the opportunistic fungal infection has been on the increase. This is mainly associated with antifungal resistance and the restricted number of available antifungal drugs [1]. *Candida* species are important opportunistic fungi in the humanbody, mainly colonizing the skin and mucous membrane such as oral cavity, gastrointestinal tract, vagina and so on. It does not cause disease in a healthy body. However, when the body's immune system declines, the extensive use of antibiotics, immunosuppressive agents, hormones, chemotherapy and other drugs, the increasing incidence of leukopenia, tumor incidence, HIV infection, diabetes and other diseases, the widespread use of invasive procedures like indwelling catheters, stapler and artificial valves, the large-scale organ transplants carried out, the increasing number of elderly patients, etc. [1,2], can lead to superficial fungal diseases and invasive candidiasis.

Currently, there are many species known to cause invasive or superficial candidiasis. And more than 90% candidiasis are caused by *Candida albicans*, *Candida glabrata*, *Candida parapsilosis*, *Candida tropicalis and Candida krusei* [3]. The most common pathogen of *Candida* spp. is *C*.*albicans*. In recent years, the incidence of non-albicans *Candida* spp. such as *C*.*glabrata*, *C*.*tropicalis*, *C*.*krusei*, *C*.*parapsilosis*has increased significantly [4,5]. A recent study conducted by Costa *et al*. showed that about 35% to 65% of all candidiasis were caused by non-albicans *Candida* spp. [6]. In North America, Horn *et al*. reported the incidence of non-albicans *Candida* spp. was 54.4%, while *C*.*albicans* was 45.6% [7]. In Argentina, Cornistein *et al*. [8] observed that *C*.*albicans* was responsible for 43.3% of all clinical specimens, whereas non-albicans *Candida* spp. were about 56.7%. Thus, *C*.*albicans* is the most common pathogenic *Candida* species, and the incidence of non-albicans *Candida* species is also on the rise.

Azole is one of the most common anti-*Candida* drugs, including fluconazole, itraconazole, posaconazole, and voriconazole. The common mechanisms of *Candida* resistant to azoles include changes in target enzyme and upregulation of multidrug resistance protein (MDR) and so on. The target enzyme of azoles is 14α-lanosterol demethylase (14-DM), encoded by *ERG11* gene. *ERG11* gene overexpression or mutations have been reported to be involved in the resistance formation of *C*.*albicans*, *C*.*glabrata*, *and C*.*tropicalis*. MDR proteins are kind of efflux pump transporters, including the ATP-binding cassette transporter family (ABC) and the major facilitator super family (MFS). In *C*.*albicans*, the efflux pump genes associated with azole resistance include *CDR1*, *CDR2*, and *MDR1* [9]; in *C*.*glabrata*, the resistance genes involved *CDR1*, *CDR2* and *SNQ2* [10] while in *C*.*tropicalis*, the azole resistance related efflux pump gene is *MDR1* [11].

At present, the study of azole resistance mechanisms of *Candida* has focused on *C*.*albicans*, *C*.*glabrata*, *C*.*tropicalis*. However, few studies talked about resistance mechanisms of *C*.*krusei*, especially resistant to itraconazole. *C*.*krusei* is naturally resistant to fluconazole. Orozco *et al*. [12] considered that fluconazole resistance in *C*.*krusei* appeared to be mediated predominantly by a reduced susceptibility of 14α-demethylase to this drug. Katiyar *et al*. [13] reported that *C*.*krusei* has found two genes encoding efflux pumps, *ABC1*, and *ABC2*. They thought that *ABC1* encoded efflux pump played a major role in innate fluconazole resistance of *C*.*krusei*, while *ABC2* gene expression was insignificant. However, Lamping *et al*. [14] and Guinea *et al*. [15] considered that it was mainly the low affinity of 14-DM with fluconazole together with the constitutive but low level of expression of the multidrug efflux pump Abc1p that were responsible for the innate *C*.*krusei* resistance to fluconazole.

To date, the mechanism of acquired drug resistance to azoles of *C*.*krusei* is still unclear. The purpose of the present study is to explore the main molecular mechanisms responsible for this species' resistance to itraconazole. Here, *ERG11* gene of 16 *C*.*krusei* clinical isolates were amplified by polymerase chain reaction (PCR), and their nucleotide sequences were determined to detect point mutations. Meanwhile, *ERG11*, *ABC1*, and *ABC2* genes were determined by quantitative real-time reverse transcription polymerase chain reaction (qRT-PCR) for their expression in itraconazole-resistant, itraconazole-susceptible dose dependent and itraconazole-susceptible *C*.*krusei* at the mRNA level.

## Materials and Methods

### 2.1. Ethics statement

This study has been approved by the Ethics Committee of Tianjin First Central Hospital. Before collecting clinical isolates from patients, we informed them of our research purposes and signed the consent form with them. Our study is to investigate the molecular mechanisms of *Candida krusei* clinical isolates resistant to itraconazole, which not only can help fully understand the resistance mechanisms of *Candida krusei*, but also can provide new ideas and approaches to clinical treatment of fungal infections. We promised to patients that their specimens were for scientific purposes only, and the patients and specimens information were anonymous in order to protect the health, safety and privacy of patients.

### 2.2. Clinical strains

The isolates of *C*.*krusei* included in the present study were from a collection of clinical isolates recovered from Tianjin First Central Hospital and Tianjin Medical University General Hospital. They were identified using *Candida* chromogenic medium (CHROMagar, France), Vitek2 YBC identification cards (BioMérieux, France) and mass spectrometer (BioMérieux, France). They were tested for their susceptibilities to flucytosine, amphotericin B, fluconazole, itraconazole, and voriconazole by ATB FUNGUS 3 (BioMérieux, France) as recommended by the manufacturer. And all the susceptibility profiles tested by ATB FUNGUS 3 were verified by the Clinical and Laboratory Standard Institute (CLSI) broth microdilution (BMD) method.

These isolates were recovered from various body sites of 16 different patients. Among the isolates studied, there were 5 itraconazole-resistant (R) strains, 8 itraconazole-susceptible dose dependent (SDD), and 3 itraconazole-susceptible (S) isolates (Table 1). In the itraconazole-resistant group, CK10 was recovered from a patient with urinary *C*.*albican* infection two weeks ago and administrated fluconazole therapy. All 16 isolates were stored at -80°C on the filter paper.

**Table 1:** Minimal Inhibitory Concentration (MIC) and susceptibility profile of *C*.*krusei* clinical isolates.

Information of strains			MIC(μg/ml)/ Susceptibility profile				
Name	Recovery site	ITR susceptibility category	5-FC	AMB	FLC	ITR	VRC
CK1	Secretions	SDD	4/S	1/S	64/R	0.25/SDD	0.25/S
CK2	Sputum	SDD	16/SDD	1/S	16/SDD	0.5/SDD	0.5/S
CK4	Urine	SDD	16/SDD	0.5/S	16/SDD	0.25/SDD	0.25/S
CK5	Secretions	R	16/SDD	0.5/S	16/SDD	1/R	0.5/S
CK6	Urine	SDD	16/SDD	0.5/S	16/SDD	0.25/SDD	0.125/S
CK8	Secretions	SDD	4/S	1/S	64/R	0.25/SDD	0.25/S
CK9	Urine	R	16/SDD	2/SDD	64/R	1/R	0.5/S
CK10	Urine	R	16/SDD	2/SDD	16/SDD	4/R	4/R
CK11	Sputum	R	16/SDD	2/SDD	16/SDD	2/R	0.25/S
CK12	Secretions	R	4/S	0.5/S	16/SDD	1/R	0.5/S
CK13	Urine	SDD	16/SDD	2/SDD	16/SDD	0.25/SDD	0.25/S
CK14	Urine	SDD	16/SDD	2/SDD	32/SDD	0.5/SDD	0.5/S
CK15	Sputum	S	4/S	0.5/S	16/SDD	0.06/S	0.125/S
CK16	Urine	SDD	8/SDD	1/S	32/SDD	0.06/S	0.125/S
CK17	Urine	S	8/SDD	2/SDD	64/R	0.5/SDD	0.25/S
CK18	Secretions	S	16/SDD	1/S	16/SDD	0.125/S	0.25/S

CK, *Candida krusei*; S, susceptible; SDD, susceptible dose dependent; R, resistant; 5-FC, 5-flucytosine, MIC break point: S, ≤4 μg/ml; SDD, 8–16 μg/ml; R, ≥32 μg/ml; AMB, Amphotericin B, MIC break point: S, ≤1 μg/ml; SDD,2 μg/ml; R, >2 μg/ml; FLC, Fluconazole, MIC break point: S, ≤8 μg/ml; SDD, 16–32 μg/ml; R, ≥ 64 μg/ml; ITR, itraconazole, MIC break point: S, ≤0.125 μg/ml; SDD, 0.25–0.5 μg/ml; R, ≥ 1 μg/ml; VRC, voriconazole, MIC break point: S, ≤0.5 μg/ml; SDD,1 μg/ml; R, ≥2 μg/ml

### 2.3. Antifungal susceptibility testing

#### 2.3.1. ATB FUNGUS 3

Reference antifungal susceptibility testing of the study isolates was performed by ATB FUNGUS 3 according to the manufacturer’s instructions, and visually read the minimal inhibitory concentration (MIC) value [16]. There are five antigungal drugs at different concentrations: 5-flucytosine (4, 16μg/ml), amphotericin B (0.5–16μg/ml), fluconazole (1–128μg/ml), itraconazole (0.125–4μg/ml) and voriconazole (0.06–8μg/ml). Testing on the ATB FUNGUS 3 was performed simultaneously with CLSI BMD method. The susceptibility profiles to itraconazole of the *C*.*krusei* isolates were refered to Sanguinetti *et al*. [10]: susceptible whenever MIC **≤**0.125μg/ml, susceptible dosedependent whenever MIC = 0.25 to 0.5 μg/ml and resistant whenever MIC **≥**1.0μg/ml. Visual readings were performed after 24h of incubation. Quality control was ensured by testing the NCCLS-recommended strains *C*.*krusei* ATCC 6258 and *C*.*parapsilosis* ATCC 22019 [17,18].

#### 2.3.2. CLSI BMD method

CLSI BMD method was performed in accordance with CLSI M27-A3 and M27-S4 guidelines. The following drugs and concentrations were tested: 5-flucytosine (0.03–64μg/ml), amphotericin B (0.008–16μg/ml), fluconazole (0.03–64 μg/ml), itraconazole (0.03–64μg/ml) and voriconazole (0.015–16μg/ml). Quality control was also ensured by testing the NCCLS-recommended strains *C*.*krusei* ATCC 6258 and *C*.*parapsilosis* ATCC 22019 [17,18]. The susceptibility profiles conducted by the above two methods were consistent, and shown in Table 1.

### 2.4. Amplifying and sequencing analysis of *ERG11* gene

Prior to experiments, such isolates were subcultured twice on Sabouraud agar (Merck KGaA, Germany) at 37°C for 24 h to revive and ensure the purity of cultures. A single one fresh colonywas transferred to 5ml liquid YPD (yeast extract 1%, peptone 2%, and dextrose 2%) broth at 35°C, 150 rpm, until exponential growth phase. 1ml yeast cells were harvested by centrifugation at room temperature (4000 rpm, 5min), and immediately frozen in liquid nitrogen. Total genomic DNA was extracted using DNA extraction kit (Omega, USA). *ERG11* gene product (1873 bp) was amplified by PCR, and primers in Table 2 are refered to Ricardo *et al*. [19]. The PCR reaction contained: 10μl of 2×Phusion Flash PCR Master Buffer (Thermo, USA), 1μl of 10μM Primer Mix, 1μl of DNA sample, and diethylpyrocarbonate (DEPC) H_2_O up to 20 μl final volume. All reactions were performed in a GeneAmp PCR system 9700 (Applied Biosystems, USA), and reaction parameters involved an initial 1 min denaturation step at 98°C followed by 35 cycles at 98°C for 10 s, at 65°C for 20s and at 72°C for 30s, with a 1 min final extension step at 72°C. PCR products were used to run 2% agarose gel, and then sent to sequence. All the *ERG11* gene sequence results were aligned with a sequence published online (Gene accession number FJ445756) using DNAMAN software.

**Table 2:** Primers used in this study.

GenBank Accession no.	Primer Name	Primer Sequences(5’→3’)
FJ445756	*ERG11*-F for sequencing	GGTTGTTTGTTCATTTAATGTGTGT
	*ERG11*-R for sequencing	GAAGGGGGAAAGAAAGGGAA
FJ445756	*ERG11*-F for RT-PCR	ATTGCGGCCGATGTCCAGAGGTAT
	*ERG11*-R for RT-PCR	GCGCAGAGTATAAGAAAGGAATGGA
DQ903907	*ABC1*-F	GATAACCATTTCCCACATTTGAGT
	*ABC1*-R	CATATGTTGCCATGTACACTTCTG
AF250037	*ABC2*-F	CCTTTTGTTCAGTGCCAGATTG
	*ABC2*-R	GTAACCAGGGACACCAGCAA
AJ389086	*ACT1*-F	TGGGCCAAAAGGATTCTTATG
	*ACT1*-R	AGATCTTTTCCATATCATCCCAG

### 2.5. *ABC1*, *ABC2* and *ERG11* genes expression analysis

#### 2.5.1. Total RNA extraction

Total RNA was extracted as previously described in this paper. After reviving and transferring to 5ml liquid YPD broth overnight, 1ml yeast cells were harvested by centrifugation at room temperature (4000 rpm, 5 min), and immediately frozen in liquid nitrogen. After mechanical disruption of the cells with liquid nitrogen grinding, the fungal powder was collected into a 2ml Eppendorf tube, and 1ml Trizol reagent (Invitrogen, USA) was added and mixed them through a pipette. Incubate the samples for 5 minutes at room temperature. Then add 250μl chloroform to the samples. Shake the tubes vigorously with hand, and incubate the samples for 10 minutes at room temperature. Centrifuge the samples at 12,000 rpm for 15 minutes at 4°C. After centrifugation, the mixture separates into 3 aqueous phases. RNA remains in the upper colorless aqueous phase. Transfer the upper colorless liquid into a new 1.5ml Eppendorf tube, and add same volume isopropyl alcohol into the tube and mix. The the mixture was incubated for at least 4 hours at -20°C to precipitate RNA. Then centrifuge the mixture at 12,000 rpm for 15 minutes at 4°C. Remove the supernatant and wash the RNA with 1ml 75% ethanol (diluted by DEPC water). Then centrifuge the mixture at 12,000 rpm for 1 minute at 4°C. Discard the supernatant and dry the RNA. Finally, RNA was dissolved in DEPC water. RNA integrity was assessed by determination of the OD260/OD280 absorption ratio, and the integrity was considered maintained if the ratio was 1.8 to 2.0. RNA samples were resuspended in DEPC water, with the concentration adjusted to a final value of 200 ng/μl and then stored at -20°C for later use.

#### 2.5.2. Reverse transcriptase PCR (RT-PCR)

One-step RT-PCR reaction was performed with a 20μl volume containing the following reagents: 6μl of Reverse Transcriptase Premixture (Thermo,USA), 7μl of 200 ng/μl RNA sample, and DEPC water up to the final volume. RT-PCR reaction was carried out in a GeneAmp PCR system 9700 (Applied Biosystems, USA), and reaction parameters were as follows: an initial 15min step at 25°C followed by 40min step at 55°C, with a final step at 85°C for 5min. The cDNAs were kept at -20°C for later use.

#### 2.5.3. Quantitative Real-Time PCR (qRT-PCR)

Quantitative expression of the *ERG11*, *ABC1*, and *ABC2* genes was conducted by real-time RT-PCR with a LightCycler 96 system (Roche, Switzerland). For the target genes and *ACT1* reference gene, they were amplified using the following primers in Table 2 refered to Ricardo *et al*. [19]. The qRT-PCR reaction mixture contained: 7.5μl of 2×KAPA SYBR FAST Mix (Kapa, USA), 1μl of 10μM Primer Mix, 1μl of 10ng cDNA sample, and DEPC water up to the final 15μl volume. Each reaction was run in triplicate. The qRT-PCR reaction condition was subjected to an initial predegeneration step at 95°C for 30s, followed by 40 cycles of 95°C for 3s, 60°C for 30s. Fluorescence data were collected and analyzed with the LightCycler 96 software.

### 2.6. Statistical analysis

Result analysis was performed with the GraphPad Prism program (GraphPad Software, Inc. San Diego, CA). The two-tailed Student's t-test was used to analyze significant differences between gene expression displayed by the distinct *C*.*krusei* strains; P<0.05 was considered statistically significant.

## Results

### 3.1. Amplifying and sequencing analysis of *ERG11* gene

#### 3.1.1. Amplifying analysis of *ERG11* gene

Total genomic DNA of all the 16 *C*.*krusei* clinical isolates were extracted to amplify *ERG11* gene, and the PCR products were used to run 2% agarose gel electrophoresis. We observed that the entire purpose band (1873bp) clearly at the same level near 2000bp.

#### 3.1.2. Sequencing analysis of *ERG11* gene

Several *ERG11* gene mutations have been previously described to be associated with azole resistance in *C*.*albicans* [20]; therefore, *C*.*krusei ERG11* gene was sequenced in our strains.

In the present study, we found 7-point mutations aligned with the online published sequence (Gene Accession Number FJ445756). And the results are presented in Table 3. There are 6 synonymous mutations and 1 missense mutation. The 6 synonymous mutations can appear in both non-resistant and resistant strains, and all the *C*.*krusei* strains presented C642T, T1389C, and G1536C mutations. The only one missense mutation occurred at 44bp (C→T), resulting inan amino acid change from alanine to valine. However, such a missense mutation can be found in non-resistant and resistant *C*.*krusei*, suggesting that the C44T missense mutation might not be associated with azoles drug resistance of *C*.*krusei*.

**Table 3:** *ERG11* gene point mutations in *C*.*krusei* clinical isolates.

Information of strains		*ERG11* gene mutation sites						
Name	ITR susceptibility category	44	51	642	756	939	1389	1536
FJ445756		C	C	C	A	T	T	G
CK1	SDD	—	—	T	—	—	C	C
CK2	SDD	T	—	T	—	—	C	C
CK4	SDD	—	T	T	—	—	C	C
CK5	R	T	T	T	—	C	C	C
CK6	SDD	T	—	T	—	C	C	C
CK8	SDD	—	—	T	—	—	C	C
CK9	R	—	—	T	—	—	C	C
CK10	R	T	T	T	—	C	C	C
CK11	R	—	—	T	T	—	C	C
CK12	R	T	—	T	—	C	C	C
CK13	SDD	T	—	T	T	—	C	C
CK14	SDD	—	—	T	—	—	C	C
CK15	S	—	T	T	—	—	C	C
CK16	SDD	T	—	T	—	C	C	C
CK17	S	—	—	T	—	—	C	C
CK18	S	T	—	T	—	—	C	C

Note: the numbering of the nucleotides shown in table 4 starts with 1 for the A of the ATG start codon. FJ445756, GenBank Accession no. of *C*.*krusei* whose *ERG11* sequence published online, used to align with our *C*.*krusei* clinical isolates in this study.

### 3.2. *ABC1*, *ABC2* and *ERG11* genes expression analysis

#### 3.2.1. *ABC1* gene expression analysis (N = 16)

**Fig 1:**
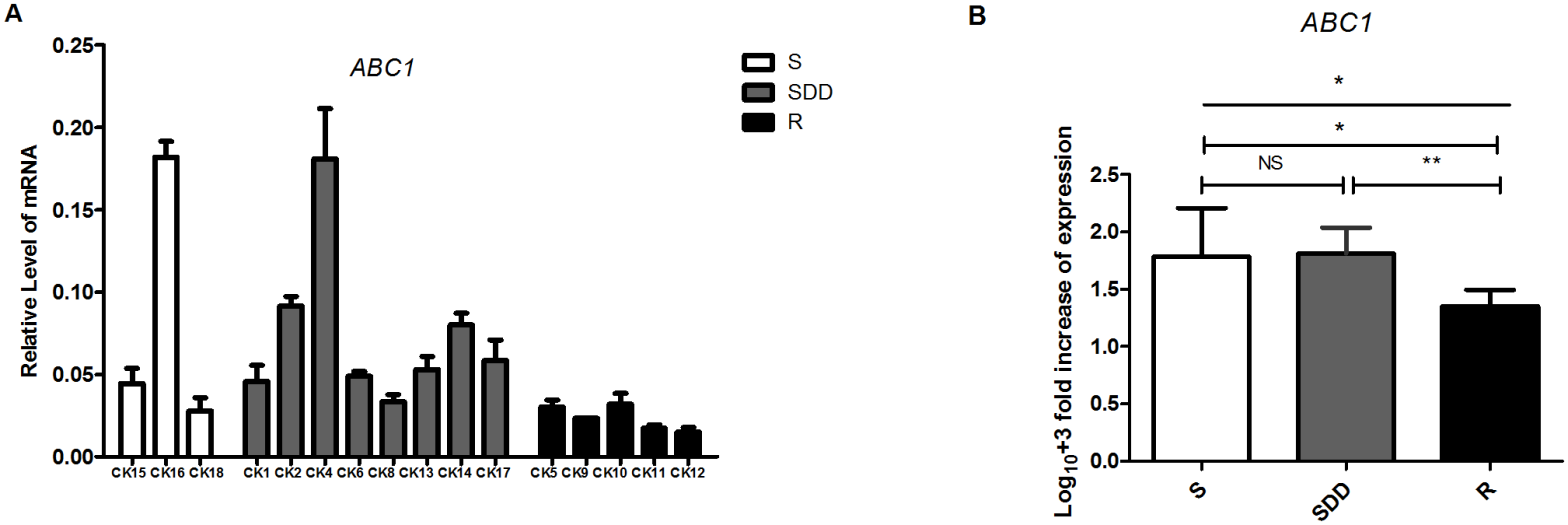
*ABC1* relative gene expression levels in three groups of *C*.*krusei* clinical isolates. (A) Relative levels of *ABC1* mRNA in all the *C*.*krusei* clinical isolates. *ABC1* gene expression levels was quantified and normalized relative to the housekeeping gene, *ACT1*; S, itraconazole-susceptible; SDD, itraconazole-susceptible dose dependent; R, itraconazole-resistant. (B) Log_10_ **+**3 fold increase of gene expression levels in three groups. (NS, no significance in SDD compared with S; *P<0.05 in R with S; **P<0.01 in R with SDD).

16 *C*.*krusei* clinical isolates *ABC1* gene mRNA relative expression levels are shown in Table 4 and Fig 1A. Individual data of *ABC1* gene mRNA relative expression in all the *C*.*krusei* is listed in S1 Table. However, in our study, the expression level of *ABC1* gene mRNA in itraconazole-resistant isolates is significantly lower than itraconazole-susceptible dose dependent and itraconazole-susceptible strains (P = 0.007 and P = 0.034 respectively, Fig 1B), and there is no significance between itraconazole-susceptible dose dependent and itraconazole-susceptible strains (P = 0.876, Fig 1B). In the itraconazole-resistant strains, a *Candida krusei* named CK10, both resistant to itraconazole and voriconazole, has the highest *ABC1* mRNA expression level in this group.

**Table 4:** *ABC1*, *ABC2* and *ERG11* genes relative level of mRNA expression in *C*.*krusei* clinical isolates.

Information of strains		Relative level of mRNA^a^(M±SD)			Log_10_+3 fold increase of expression^b^		
Name	ITR susceptibility category	*ABC1*	*ABC2*	*ERG11*	*ABC1*	*ABC2*	*ERG11*
CK1	SDD	0.0458 ± 0.0097	0.0733 ± 0.0056	0.0483 ± 0.0066	1.66	1.87	1.68
CK2	SDD	0.0918 ± 0.0056	0.1141 ± 0.0006	0.0506 ± 0.0035	1.96	2.06	1.70
CK4	SDD	0.1811 ± 0.0304	0.0729 ± 0.0161	0.0469 ± 0.0160	2.26	1.86	1.67
CK5	R	0.0304 ± 0.0041	0.0902 ± 0.0047	0.0525 ± 0.0093	1.48	1.96	1.72
CK6	SDD	0.0491 ± 0.0027	0.0506 ± 0.0058	0.0802 ± 0.0113	1.69	1.70	1.90
CK8	SDD	0.0336 ± 0.0041	0.1191 ± 0.0197	0.0955 ± 0.0089	1.53	2.08	1.98
CK9	R	0.0237 ± 0.0001	0.1419 ± 0.0600	0.1036 ± 0.0068	1.37	2.15	2.02
CK10	R	0.0319 ± 0.0067	0.2338 ± 0.0337	0.1654 ± 0.0063	1.50	2.37	2.22
CK11	R	0.0175 ± 0.0021	0.1336 ± 0.0149	0.1525 ± 0.0125	1.24	2.13	2.18
CK12	R	0.0151 ± 0.0027	0.1298 ± 0.0134	0.0875 ± 0.0118	1.18	2.11	1.94
CK13	SDD	0.0532 ± 0.0079	0.0818 ± 0.0249	0.0421 ± 0.0036	1.73	1.91	1.62
CK14	SDD	0.0801 ± 0.0071	0.0279 ± 0.0015	0.0122 ± 0.0003	1.90	1.45	1.09
CK15	S	0.0446 ± 0.0091	0.0408 ± 0.0049	0.0228 ± 0.0017	1.65	1.61	1.36
CK16	S	0.1819 ± 0.0097	0.0136 ± 0.0178	0.0207 ± 0.0015	2.26	1.13	1.32
CK17	SDD	0.0586 ± 0.0125	0.0532 ± 0.0042	0.0325 ± 0.0083	1.77	1.73	1.51
CK18	S	0.0280 ± 0.0078	0.0296 ± 0.0037	0.0305 ± 0.0049	1.45	1.47	1.48

Note:

^a^ Quantification was performed by real-time RT-PCR. The values are averages of triplicate wells. M: mean; SD: standard deviation.

^b^ The data was transformed from the values of relative level of mRNA, and obeyed the normal distribution and variance homogeneity.

#### 3.2.2. *ABC2* gene expression analysis (N = 16)

**Fig 2:**
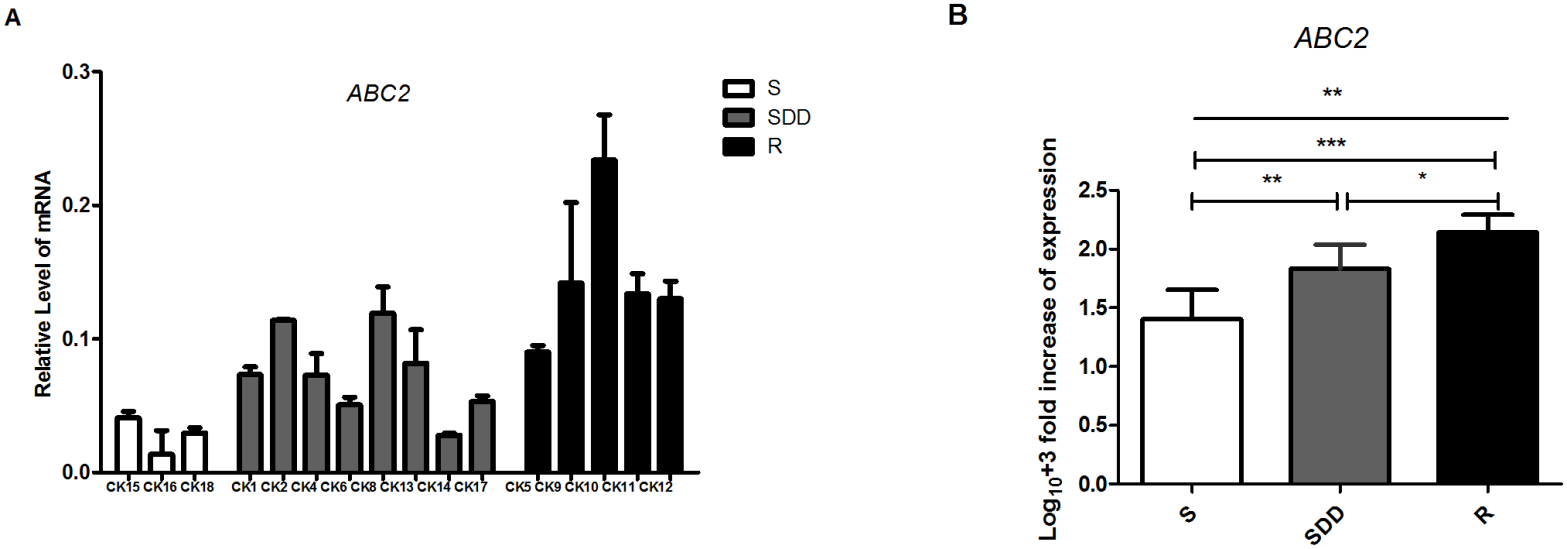
*ABC2* relative gene expression levels in three groups of *C*.*krusei* clinical isolates. (A) Relative levels of *ABC2* mRNA in all the *C*.*krusei* clinical isolates. *ABC2* gene expression levels was quantified and normalized relative to the housekeeping gene, *ACT1*; S, itraconazole-susceptible; SDD, itraconazole-susceptible dose dependent; R, itraconazole-resistant. (B) Log_10_ **+**3 fold increase of gene expression levels in three groups. (*P<0.05 in R compared with SDD; **P<0.01 in SDD with S; ***P<0.001 in R with S).

16 clinical isolates of *C*.*krusei ABC2* gene mRNA relative expression levels are presented in Table 4 and Fig 2A. Individual data of *ABC2* gene mRNA relative expression in all the *C*.*krusei* is listed in S2 Table. Our data shows that the expression level of *ABC2* gene mRNA in itraconazole-resistant isolates is significantly higher than itraconazole-susceptible dose dependent (P = 0.016, Fig 2B) and itraconazole-susceptible strains (P<0.001, Fig 2B). The mRNA level in itraconazole-susceptible dose dependent group is also significantly higher compared to itraconazole-susceptible one (P = 0.007, Fig 2B). And in the itraconazole-resistant strains, the *ABC2* mRNA expression level of CK10 is highest in this group.

#### 3.2.3. *ERG11* gene expression analysis (N = 16)

**Fig 3:**
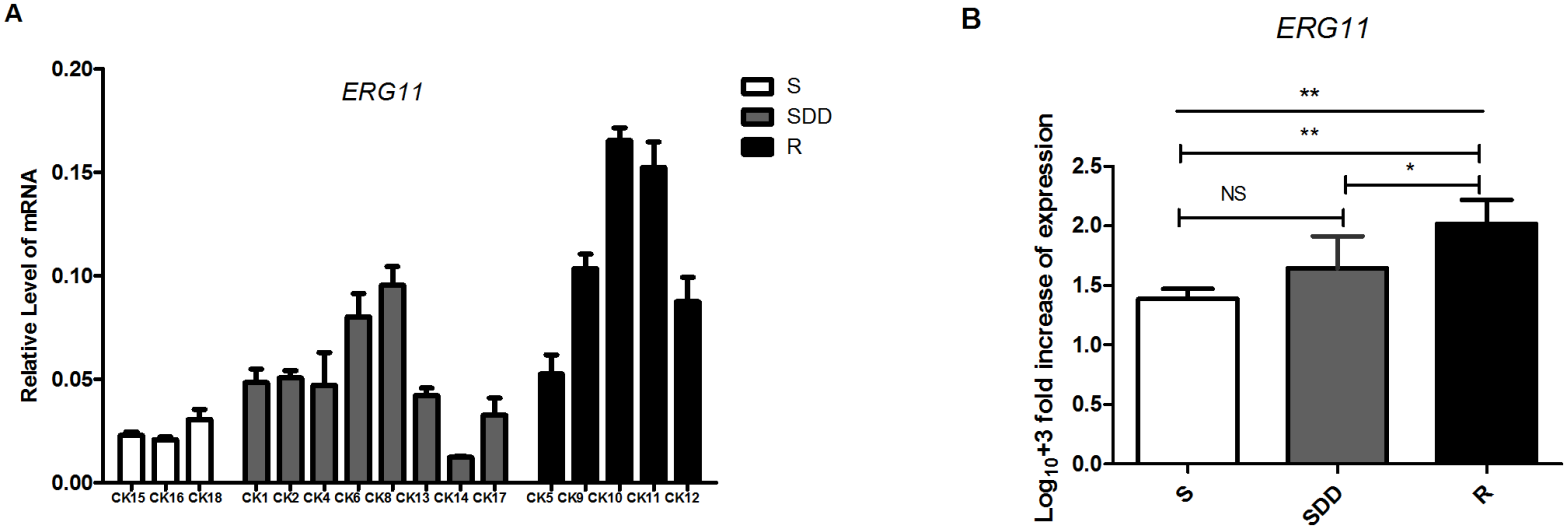
*ERG11* relative gene expression levels in three groups of *C*.*krusei* clinical isolates. (A) Relative levels of *ERG11* mRNA in all the *C*.*krusei* clinical isolates. *ERG11* gene expression levels was quantified and normalized relative to the housekeeping gene, *ACT1*; S, itraconazole-susceptible; SDD, itraconazole-susceptible dose dependent; R, itraconazole-resistant. (B) Log_10_ **+**3 fold increase of gene expression levels in three groups. (NS, no significance in SDD compared with S; *P<0.05 in R with SDD; **P<0.01 in R with S).

16 *C*.*krusei* clinical isolates *ERG11* gene mRNA relative expression levels are shown in Table 4 and Fig 3A. Individual data of *ERG11* gene mRNA relative expression in all the *C*.*krusei* is listed in S3 Table. Our data shows that the expression level of *ERG11* gene mRNA in itraconazole-resistant isolates is also significantly higher than itraconazole-susceptible dose dependent (P = 0.015, Fig 3B) and itraconazole-susceptible strains (P = 0.002, Fig 3B). But there is insignificant between itraconazole-susceptible dose dependent isolates and itraconazole-susceptible ones (P = 0.12, Fig 3B). However, in the itraconazole-resistant strains, the *ERG11* mRNA expression level of CK10 is still the highest in this group.

## Discussion

Azole antifungal agents are ergosterol biosynthesis inhibitors. On one hand, azole combines with target enzyme 14-DM to inhibit ergosterol biosynthesis. On the other, it can lead to accumulation of toxic steroid through bypass. These two ways synergize to exert antifungal effect on *Candida*. However, due to its inhibition effect on *Candida*, it can cause the emergence of drug resistance after a long course of treatment or repeated dose administration. The common mechanisms of *Candida* resistant to azoles include changes in target enzyme and upregulation of multidrug resistance protein (MDR). These could occur through (1) increased *ERG11* gene overexpression, which can cause target enzyme to be produced more, leading to a higher concentration of azoles to block the biosynthesis of 14-DM; (2) *ERG11* gene existed mutations, which can cause structural change of 14-DM, resulting in affinity decline between azoles and 14-DM; (3) efflux pumps of MDR upregulation, which can decrease the azole concentration within the cell, making it unable to inhibit fungi cell effectively, thus leading to the emergence of drug resistance. To date, there are two kinds of efflux transporters associated with *C*.*krusei* resistance: Abc1p and Abc2p, encoded by the gene *ABC1* and *ABC2* respectively.

Currently, studies on azole resistance mechanisms of *Candida* mainly focused on *C*.*albicans*, *C*.*glabrata*, and *C*.*tropicalis*. However, there are few researches discussing the resistance mechanisms of *C*.*krusei*. In addition, the mechanisms of *Candida* resistant to different azoles also showed distinctly. Research results suggested that *ERG11*, *ABC1* and *ABC2* genes were associated with azole resistance of *C*.*krusei* [13, 21]. However, the strains studied by researchers were mostly from the same patient at a different stage, with strictly and genetically matched fluconazole-resistant and fluconazole-sensitive ones. In this case, the number of patients or the number of strains is always limited. The strains used in other studies were from lab-induced resistant ones. However, in clinical practice, it is always difficult to obtain strictly and genetically matched isolates. In addition, whether the resistance mechanisms of lab-induced strains are similar to clinical isolates from different patients is still unclear. As we all know, the resistance mechanisms of strictly and genetically paired strains cannot completely explain the mechanisms of azole resistance in unmatched isolates. Therefore, it is very important to study the resistance mechanisms of unmatched isolates of *C*.*krusei* from different patients, which can help fully understand the mechanisms of resistance formation.

In the present study, we recovered 16 *C*.*krusei* clinical isolates from different patients and divided them into three groups according to their susceptibility to itraconazole. There were 5 strains in the itraconazole-resistant group, 8 in the itraconazole-susceptible dose dependent group, and 3 in the itraconazole-susceptible one. Here, to explore the relation between *ERG11*, *ABC1* and *ABC2* genes and resistance formation of itraconazole in clinical isolates of *C*.*krusei*, we used PCR to amplify *ERG11* gene and sequenced it in order to find new mutations. Then, we performed qRT-PCR to detect the differences of the resistant gene expression levels in the three groups.

In order to fully understand *ERG11* gene mutation of *C*.*krusei*, we amplified the PCR product containing the whole open reading frame of *ERG11* gene and sent it to sequence in our study. The PCR product is about 1873bp, including 1587bp of *ERG11* gene, which encodes 529 amino acids. Our data showed that there were 7-point mutations existing in *ERG11* gene of *C*.*krusei*, including 6 synonymous mutations (C51T, C642T, A756T, T939C, T1389C, and G1536C) and 1 missense mutation (C44T). The only missense mutation can result in the 15th amino acid changing from alanine to valine. However, such a missense mutation can be found in the three groups, indicating that C44T may not be involved in itraconazole resistance of *C*.*krusei*. Moreover, in our study, all the non-resistant and resistant *C*.*krusei* strains presented C642T, T1389C, G1536C mutations. Ricardo *et al*. [19] also reported that T1389C mutation occurred in all the experimental strains. Besides, T939C mutation have been reported by Tavakoli *et al*. [21]. In addition, all the point mutations found in our experiment are consistent with *ERG11* gene single nucleotide polymorphism (SNP) sites reported by Lamping [14] except for C51T, a new synonymous mutation which has not been reported before.

However, T418C missense mutation reported by Ricardo [19] has not been found in our study, further illustrating the mechanism differences between in vivo or in vitro induced resistance and drug resistance in clinical isolates of *C*.*krusei* from different persons. Previous researches [22,23] reported that, *ERG11* gene missen mutation was associated with *C*.*albicans* and *C*.*tropicalis* azole resistance, which can cause chages in amino acid sequence resulting in structural change of target enzyme 14-DM. This can lead to decreased affinity with fluconazole and the emergence of resistance. In our view, C44T missense mutation may occur outside the active site of 14-DM, which doesn’t affect the interaction of azole and 14-DM or a single missense mutation is not enough to change the affinity of the 14-DM to azole. On the other hand, the target enzyme structure of *C*.*krusei* may be a lot different from other *Candida*, and its *ERG11* gene polymorphism is more complicated.

Our study also found that, *ERG11* and *ABC2* genes mRNA expression levels of itraconazole-resistant group in *C*.*krusei* clinical isolates were significantly higher than itraconazole non-resistant strains. And the mRNA levels of *ABC2* gene expression in the isolates appeared to increase with the decrease of susceptibility to itraconazole. While *ABC1* gene mRNA expression level in resistant group was significantly lower than itraconazole non-resistant groups. In addition, an isolate in the resistant group named CK10, recovered from a patient pre-exposed to fluconazole, which was resistant to itraconazole and voriconazole, has its *ERG11*, *ABC1* and *ABC2* genes mRNA expression levels as the highest in this group. We speculate that the resistance mechanisms of itraconazole and voriconazole in *C*.*krusei* clinical isolates may be different. However, Lamping *et al*. [14] considered that Abc1p played a major role in innate fluconazole resistance of *C*.*krusei*. Similar findings were previously described by Ricardo *et al*. [19] for *C*.*krusei*. They considered that *ABC2* can be more rapidly activated than *ABC2* when exposure to voriconazole [19]. When involving long term voriconazole tolerance, Abc1p efflux pump seemed to be more efficient in antifungal expelling and played a late role in the development of resistance. And their data also showed that, “*ERG11* gene overexpression seems to be relevant to the development of voriconazole resistance only at an early stage, as an initial adaptation mechanism. Later on, other distinct mechanisms such as the acquisition of point mutations may take the lead [19].” Venkateswarlu *et al*. [24] reported that the accumulation of itraconazole in resistant *C*.*krusei* was less than that in sensitive strains, but the two strains showing different sensitivity to itraconazole were both highly resistant to fluconazole, suggesting that the resistance mechanism between fluconazole and itraconazole in *C*.*krusei* may vary from each other. They thought that *C*.*krusei* resistant to itraconazole was due to reduced drug accumulation in fungal cells and guessed that there may exist one or more efflux pumps contributing to *C*.*krusei* itraconazole resistance, which can be well explained in our study. Our data presented that Abc2p may play a more important role in *C*.*krusei* itraconazole resistance, instead of Abc1p.

In conclusion, *ERG11*, *ABC1* and *ABC2* genes of *C*.*krusei* clinical isolates play different roles in the development of itraconazole resistance. However, in this study, we conclude that *ERG11* and *ABC2* genes upregulation contribute mostly to itraconazole resistance of *C*.*krusei* clinical isolates while *ERG11* gene mutations and *ABC1* gene may not be associated with the development of itraconazole resistance in our study. Due to our limited number of experimental strains, it is unreasonable to rule out the participation of *ERG11* mutations in the emergence of resistance. Whether *ERG11* mutations affect *ERG11* gene expression is still unknown, and this needs to be further studied.

## Supporting Information

S1 Table Individual data of *ABC1* gene mRNA relative expression in all the *C*.*krusei* exported from LightCycler 96 softwware. Ratio: the target gene *ABC1* mRNA levels relative to the reference gene *ACT1*.
(PDF)

S2 Table Individual data of *ABC2* gene mRNA relative expression in all the *C*.*krusei* exported from LightCycler 96 softwware. Ratio: the target gene *ABC2* mRNA levels relative to the reference gene *ACT1*.
(PDF)

S3 Table Individual data of *ERG11* gene mRNA relative expression in all the *C*.*krusei* exported from LightCycler 96 softwware. Ratio: the target gene *ERG11* mRNA levels relative to the reference gene *ACT1*.
(PDF)
