# Supplementary material for: Overexpression of Both ERG11 and ABC2 Genes Might Be Responsible for Itraconazole Resistance in Clinical Isolates of Candida krusei
Source: PLoS One. 2015 Aug 26;10(8):e0136185. doi: 10.1371/journal.pone.0136185 (PMC4550294; doi:10.1371/journal.pone.0136185)
Supplement: S1 Table — Ratio: the target gene ABC1 mRNA levels relative to the reference gene ACT1. (PDF) [file pone.0136185.s001.pdf]

Position	Sample Name	Gene Name	Cq	Cq Mean	Cq Error	Gene Type	Replicate Group	Ratio	Ratio Error	Dye	Failure	Slope	EPF
A1	CK1	ACT1	18.83	18.7	0.23	Reference	A1	-	-	SYBR Green I	None	3.31	7.02
A2	CK1	ACT1	18.83	18.7	0.23	Reference	A1	-	-	SYBR Green I	None	3.41	7.02
A3	CK1	ACT1	18.44	18.7	0.23	Reference	A1	-	-	SYBR Green I	None	3.44	7.05
A4	CK2	ACT1	16.2	16.4	0.23	Reference	A4	-	-	SYBR Green I	None	2.97	6.85
A5	CK2	ACT1	16.36	16.4	0.23	Reference	A4	-	-	SYBR Green I	None	2.96	6.8
A6	CK2	ACT1	16.65	16.4	0.23	Reference	A4	-	-	SYBR Green I	None	2.84	6.76
A7	CK4	ACT1	16.91	16.73	0.33	Reference	A7	-	-	SYBR Green I	None	3.41	7.12
A8	CK4	ACT1	16.35	16.73	0.33	Reference	A7	-	-	SYBR Green I	None	2.99	6.89
A9	CK4	ACT1	16.92	16.73	0.33	Reference	A7	-	-	SYBR Green I	None	3.09	6.91
A10	CK5	ACT1	18.66	18.8	0.2	Reference	A10	-	-	SYBR Green I	None	3.05	6.94
A11	CK5	ACT1	18.94	18.8	0.2	Reference	A10	-	-	SYBR Green I	None	3.28	6.94
A12	CK5	ACT1	-	-	-	Reference	A10	-	-	SYBR Green I	Failure	0.1	0.43
B1	CK1	ABC1	23.55	23.17	0.33	Target	B1	3.47E-02	5.41E-03	SYBR Green I	None	4.11	7.02
B2	CK1	ABC1	22.95	23.17	0.33	Target	B1	5.26E-02	8.20E-03	SYBR Green I	None	4.01	6.87
B3	CK1	ABC1	23.02	23.17	0.33	Target	B1	5.01E-02	7.81E-03	SYBR Green I	None	3.86	6.8
B4	CK2	ABC1	19.95	19.85	0.09	Target	B4	8.56E-02	1.35E-02	SYBR Green I	None	3.44	6.68
B5	CK2	ABC1	19.82	19.85	0.09	Target	B4	9.36E-02	1.48E-02	SYBR Green I	None	3	6.36
B6	CK2	ABC1	19.78	19.85	0.09	Target	B4	9.63E-02	1.52E-02	SYBR Green I	None	3	6.46
B7	CK4	ABC1	19.5	19.21	0.26	Target	B7	1.46E-01	3.31E-02	SYBR Green I	None	3.87	7.09
B8	CK4	ABC1	19.03	19.21	0.26	Target	B7	2.03E-01	4.58E-02	SYBR Green I	None	3.95	7.09
B9	CK4	ABC1	19.09	19.21	0.26	Target	B7	1.94E-01	4.40E-02	SYBR Green I	None	3.93	7.09
B10	CK5	ABC1	23.76	23.85	0.2	Target	B10	3.21E-02	4.41E-03	SYBR Green I	None	3.58	6.65
B11	CK5	ABC1	24.08	23.85	0.2	Target	B10	2.57E-02	3.53E-03	SYBR Green I	None	3.62	6.65
B12	CK5	ABC1	23.71	23.85	0.2	Target	B10	3.33E-02	4.57E-03	SYBR Green I	None	3.47	6.56
C1	CK6	ACT1	18.48	18.61	0.17	Reference	C1	-	-	SYBR Green I	None	3.17	6.86
C2	CK6	ACT1	18.55	18.61	0.17	Reference	C1	-	-	SYBR Green I	None	3.31	6.88
C3	CK6	ACT1	18.8	18.61	0.17	Reference	C1	-	-	SYBR Green I	None	3.13	7
C4	CK8	ACT1	18.53	18.47	0.05	Reference	C4	-	-	SYBR Green I	None	2.56	6.37
C5	CK8	ACT1	18.45	18.47	0.05	Reference	C4	-	-	SYBR Green I	None	2.73	6.57
C6	CK8	ACT1	18.43	18.47	0.05	Reference	C4	-	-	SYBR Green I	None	2.98	6.7
C7	CK9	ACT1	18.65	18.76	0.18	Reference	C7	-	-	SYBR Green I	None	3.18	6.89
C8	CK9	ACT1	18.96	18.76	0.18	Reference	C7	-	-	SYBR Green I	None	3.28	7.02
C9	CK9	ACT1	18.66	18.76	0.18	Reference	C7	-	-	SYBR Green I	None	3.27	6.92
C10	CK10	ACT1	18.29	18.35	0.11	Reference	C10	-	-	SYBR Green I	None	3.09	6.81
C11	CK10	ACT1	18.47	18.35	0.11	Reference	C10	-	-	SYBR Green I	None	2.83	6.66
C12	CK10	ACT1	18.28	18.35	0.11	Reference	C10	-	-	SYBR Green I	None	2.63	6.49
D1	CK6	ABC1	22.99	22.96	0.08	Target	D1	4.80E-02	5.60E-03	SYBR Green I	None	3.8	6.89
D2	CK6	ABC1	23.02	22.96	0.08	Target	D1	4.70E-02	5.49E-03	SYBR Green I	None	3.78	6.75
D3	CK6	ABC1	22.87	22.96	0.08	Target	D1	5.22E-02	6.09E-03	SYBR Green I	None	3.75	6.79
D4	CK8	ABC1	23.31	23.37	0.18	Target	D4	3.49E-02	1.28E-03	SYBR Green I	None	2.87	6.42
D5	CK8	ABC1	23.23	23.37	0.18	Target	D4	3.69E-02	1.35E-03	SYBR Green I	None	3.34	6.62
D6	CK8	ABC1	23.58	23.37	0.18	Target	D4	2.90E-02	1.06E-03	SYBR Green I	None	3.43	6.56
D7	CK9	ABC1	24.15	24.44	0.49	Target	D7	2.38E-02	2.91E-03	SYBR Green I	None	1.67	5.47
D8	CK9	ABC1	24.16	24.44	0.49	Target	D7	2.36E-02	2.89E-03	SYBR Green I	None	3.71	6.66
D9	CK9	ABC1	24.16	24.44	0.49	Target	D7	2.36E-02	2.89E-03	SYBR Green I	None	3.56	6.62
D10	CK10	ABC1	23.63	23.34	0.3	Target	D10	2.57E-02	1.90E-03	SYBR Green I	None	3.72	6.83
D11	CK10	ABC1	23.35	23.34	0.3	Target	D10	3.12E-02	2.31E-03	SYBR Green I	None	3.4	6.62
D12	CK10	ABC1	23.03	23.34	0.3	Target	D10	3.89E-02	2.89E-03	SYBR Green I	None	3.08	6.41
E1	CK11	ACT1	18.28	18.26	0.1	Reference	E1	-	-	SYBR Green I	None	2.61	6.56
E2	CK11	ACT1	18.16	18.26	0.1	Reference	E1	-	-	SYBR Green I	None	2.7	6.62
E3	CK11	ACT1	18.35	18.26	0.1	Reference	E1	-	-	SYBR Green I	None	2.83	6.77
E4	CK12	ACT1	17.19	16.94	0.22	Reference	E4	-	-	SYBR Green I	None	2.91	6.71
E5	CK12	ACT1	16.87	16.94	0.22	Reference	E4	-	-	SYBR Green I	None	2.66	6.62
E6	CK12	ACT1	16.77	16.94	0.22	Reference	E4	-	-	SYBR Green I	None	2.66	6.62
E7	CK13	ACT1	17.6	17.56	0.05	Reference	E7	-	-	SYBR Green I	None	2.95	6.86
E8	CK13	ACT1	17.5	17.56	0.05	Reference	E7	-	-	SYBR Green I	None	2.58	6.52
E9	CK13	ACT1	17.57	17.56	0.05	Reference	E7	-	-	SYBR Green I	None	2.53	6.38
E10	CK14	ACT1	17	17.13	0.34	Reference	E10	-	-	SYBR Green I	None	2.81	6.66
E11	CK14	ACT1	16.87	17.13	0.34	Reference	E10	-	-	SYBR Green I	None	2.78	6.63
E12	CK14	ACT1	17.52	17.13	0.34	Reference	E10	-	-	SYBR Green I	None	2.88	6.74
F1	CK11	ABC1	24.18	24.1	0.17	Target	F1	1.65E-02	8.06E-03	SYBR Green I	None	3.26	6.49
F2	CK11	ABC1	24.21	24.1	0.17	Target	F1	1.61E-02	7.89E-03	SYBR Green I	None	3.29	6.56
F3	CK11	ABC1	23.9	24.1	0.17	Target	F1	2.00E-02	9.78E-03	SYBR Green I	None	3.43	6.61
F4	CK12	ABC1	22.9	23.01	0.28	Target	F4	1.61E-02	2.45E-03	SYBR Green I	None	3.2	6.46
F5	CK12	ABC1	23.33	23.01	0.28	Target	F4	1.20E-02	1.82E-03	SYBR Green I	None	3.33	6.64
F6	CK12	ABC1	22.81	23.01	0.28	Target	F4	1.71E-02	2.61E-03	SYBR Green I	None	3.2	6.45
F7	CK13	ABC1	21.75	21.8	0.22	Target	F7	5.47E-02	1.94E-03	SYBR Green I	None	3.39	6.61
F8	CK13	ABC1	22.04	21.8	0.22	Target	F7	4.47E-02	1.59E-03	SYBR Green I	None	3.31	6.6
F9	CK13	ABC1	21.61	21.8	0.22	Target	F7	6.02E-02	2.14E-03	SYBR Green I	None	2.9	6.28
F10	CK14	ABC1	20.89	20.78	0.13	Target	F10	7.38E-02	1.76E-02	SYBR Green I	None	3.27	6.77
F11	CK14	ABC1	20.8	20.78	0.13	Target	F10	7.86E-02	1.87E-02	SYBR Green I	None	3.3	6.64
F12	CK14	ABC1	20.64	20.78	0.13	Target	F10	8.78E-02	2.09E-02	SYBR Green I	None	3.36	6.69
G1	CK15	ACT1	18.98	19.19	0.21	Reference	G1	-	-	SYBR Green I	None	3.02	6.81
G2	CK15	ACT1	19.39	19.19	0.21	Reference	G1	-	-	SYBR Green I	None	3	6.76
G3	CK15	ACT1	19.19	19.19	0.21	Reference	G1	-	-	SYBR Green I	None	2.86	6.61
G4	CK16	ACT1	16.11	16.01	0.13	Reference	G4	-	-	SYBR Green I	None	2.84	6.75
G5	CK16	ACT1	15.86	16.01	0.13	Reference	G4	-	-	SYBR Green I	None	2.89	6.7
G6	CK16	ACT1	16.07	16.01	0.13	Reference	G4	-	-	SYBR Green I	None	3.03	6.87
G7	CK17	ACT1	18.61	18.53	0.08	Reference	G7	-	-	SYBR Green I	None	3.17	7
G8	CK17	ACT1	18.53	18.53	0.08	Reference	G7	-	-	SYBR Green I	None	3	6.86
G9	CK17	ACT1	18.46	18.53	0.08	Reference	G7	-	-	SYBR Green I	None	3.33	6.88
G10	CK18	ACT1	18.34	18.26	0.23	Reference	G10	-	-	SYBR Green I	None	2.74	6.61
G11	CK18	ACT1	18.44	18.26	0.23	Reference	G10	-	-	SYBR Green I	None	2.81	6.76
G12	CK18	ACT1	18	18.26	0.23	Reference	G10	-	-	SYBR Green I	None	3.13	6.85
H1	CK15	ABC1	24.02	23.69	0.3	Target	H1	3.51E-02	4.99E-03	SYBR Green I	None	3.36	6.5
H2	CK15	ABC1	23.64	23.69	0.3	Target	H1	4.57E-02	6.49E-03	SYBR Green I	None	3.3	6.53
H3	CK15	ABC1	23.42	23.69	0.3	Target	H1	5.32E-02	7.56E-03	SYBR Green I	None	2.82	6.15
H4	CK16	ABC1	18.45	18.47	0.08	Target	H4	1.85E-01	1.72E-02	SYBR Green I	None	2.89	6.39
H5	CK16	ABC1	18.41	18.47	0.08	Target	H4	1.90E-01	1.77E-02	SYBR Green I	None	3.41	6.73
H6	CK16	ABC1	18.56	18.47	0.08	Target	H4	1.71E-01	1.59E-02	SYBR Green I	None	3.68	6.93
H7	CK17	ABC1	22.78	22.65	0.29	Target	H7	5.27E-02	2.74E-03	SYBR Green I	None	3.52	6.86
H8	CK17	ABC1	22.85	22.65	0.29	Target	H7	5.02E-02	2.61E-03	SYBR Green I	None	3.6	6.67
H9	CK17	ABC1	22.31	22.65	0.29	Target	H7	7.30E-02	3.80E-03	SYBR Green I	None	3.8	6.82
H10	CK18	ABC1	23.6	23.5	0.3	Target	H10	2.80E-02	4.38E-03	SYBR Green I	None	3.43	6.56
H11	CK18	ABC1	23.16	23.5	0.3	Target	H10	3.35E-02	5.36E-03	SYBR Green I	None	3.32	6.49
H12	CK18	ABC1	23.74	23.5	0.3	Target	H10	2.24E-02	3.58E-03	SYBR Green I	None	3.66	6.81
