# Supplementary material for: Overexpression of Both ERG11 and ABC2 Genes Might Be Responsible for Itraconazole Resistance in Clinical Isolates of Candida krusei
Source: PLoS One. 2015 Aug 26;10(8):e0136185. doi: 10.1371/journal.pone.0136185 (PMC4550294; doi:10.1371/journal.pone.0136185)
Supplement: S2 Table — Ratio: the target gene ABC2 mRNA levels relative to the reference gene ACT1. (PDF) [file pone.0136185.s002.pdf]

Position	Sample Name	Gene Name	Cq	Cq Mean	Cq Error	Gene Type	Replicate Group	Ratio	Ratio Erro	Dye	Failure	Slope	EPF
A1	CK1	ACT1	16.23	16.07	0.2	Reference	A1	-	-	SYBR Green I	None	3.01	6.92
A2	CK1	ACT1	15.85	16.07	0.2	Reference	A1	-	-	SYBR Green I	None	2.8	6.73
A3	CK1	ACT1	16.12	16.07	0.2	Reference	A1	-	-	SYBR Green I	None	2.7	6.67
A4	CK2	ACT1	18.14	18.3	0.15	Reference	A4	-	-	SYBR Green I	None	3.13	6.87
A5	CK2	ACT1	18.44	18.3	0.15	Reference	A4	-	-	SYBR Green I	None	3.15	6.89
A6	CK2	ACT1	18.33	18.3	0.15	Reference	A4	-	-	SYBR Green I	None	3.29	6.96
A7	CK4	ACT1	19.13	19.12	0.12	Reference	A7	-	-	SYBR Green I	None	3.18	6.95
A8	CK4	ACT1	19	19.12	0.12	Reference	A7	-	-	SYBR Green I	None	3.06	6.93
A9	CK4	ACT1	19.24	19.12	0.12	Reference	A7	-	-	SYBR Green I	None	3.15	7.12
A10	CK5	ACT1	17.42	17.41	0.04	Reference	A10	-	-	SYBR Green I	None	2.8	6.8
A11	CK5	ACT1	17.44	17.41	0.04	Reference	A10	-	-	SYBR Green I	None	2.79	6.96
A12	CK5	ACT1	17.37	17.41	0.04	Reference	A10	-	-	SYBR Green I	None	2.86	6.9
B1	CK1	ABC2	19.77	19.84	0.11	Target	B1	7.68E-02	1.04E-02	SYBR Green I	None	3.49	6.83
B2	CK1	ABC2	19.78	19.84	0.11	Target	B1	7.62E-02	1.03E-02	SYBR Green I	None	3.32	6.66
B3	CK1	ABC2	19.97	19.84	0.11	Target	B1	6.68E-02	9.06E-03	SYBR Green I	None	3.04	6.47
B4	CK2	ABC2	21.44	21.43	0.01	Target	B4	1.14E-01	1.20E-02	SYBR Green I	None	3.66	6.8
B5	CK2	ABC2	21.44	21.43	0.01	Target	B4	1.14E-01	1.20E-02	SYBR Green I	None	3.68	6.8
B6	CK2	ABC2	21.43	21.43	0.01	Target	B4	1.15E-01	1.20E-02	SYBR Green I	None	3.69	6.81
B7	CK4	ABC2	23.18	22.92	0.31	Target	B7	6.00E-02	2.94E-02	SYBR Green I	None	3.48	6.62
B8	CK4	ABC2	23	22.92	0.31	Target	B7	6.79E-02	3.33E-02	SYBR Green I	None	3.56	6.72
B9	CK4	ABC2	22.58	22.92	0.31	Target	B7	9.09E-02	4.46E-02	SYBR Green I	None	3.64	6.75
B10	CK5	ABC2	20.86	20.88	0.08	Target	B10	9.12E-02	2.27E-02	SYBR Green I	None	3.12	6.57
B11	CK5	ABC2	20.81	20.88	0.08	Target	B10	9.44E-02	2.35E-02	SYBR Green I	None	3.04	6.41
B12	CK5	ABC2	20.96	20.88	0.08	Target	B10	8.51E-02	2.12E-02	SYBR Green I	None	3.28	6.61
C1	CK6	ACT1	17	16.86	0.15	Reference	C1	-	-	SYBR Green I	None	3.24	7.03
C2	CK6	ACT1	16.71	16.86	0.15	Reference	C1	-	-	SYBR Green I	None	3.04	6.95
C3	CK6	ACT1	16.86	16.86	0.15	Reference	C1	-	-	SYBR Green I	None	2.86	6.78
C4	CK8	ACT1	18.76	18.78	0.21	Reference	C4	-	-	SYBR Green I	None	3.03	6.82
C5	CK8	ACT1	19	18.78	0.21	Reference	C4	-	-	SYBR Green I	None	3.09	6.93
C6	CK8	ACT1	18.58	18.78	0.21	Reference	C4	-	-	SYBR Green I	None	3.03	6.76
C7	CK9	ACT1	19.03	18.85	0.19	Reference	C7	-	-	SYBR Green I	None	2.61	6.59
C8	CK9	ACT1	18.65	18.85	0.19	Reference	C7	-	-	SYBR Green I	None	2.88	6.85
C9	CK9	ACT1	18.87	18.85	0.19	Reference	C7	-	-	SYBR Green I	None	2.98	6.93
C10	CK10	ACT1	19.14	19.18	0.04	Reference	C10	-	-	SYBR Green I	None	3.16	6.92
C11	CK10	ACT1	19.22	19.18	0.04	Reference	C10	-	-	SYBR Green I	None	3.27	7.06
C12	CK10	ACT1	19.19	19.18	0.04	Reference	C10	-	-	SYBR Green I	None	3.03	6.85
D1	CK6	ABC2	20.99	21.17	0.16	Target	D1	5.70E-02	5.73E-03	SYBR Green I	None	3.44	6.71
D2	CK6	ABC2	21.2	21.17	0.16	Target	D1	4.93E-02	4.95E-03	SYBR Green I	None	3.48	6.73
D3	CK6	ABC2	21.31	21.17	0.16	Target	D1	4.57E-02	4.59E-03	SYBR Green I	None	3.61	6.72
D4	CK8	ABC2	21.87	21.86	0.17	Target	D4	1.19E-01	1.60E-02	SYBR Green I	None	3.61	6.72
D5	CK8	ABC2	21.69	21.86	0.17	Target	D4	1.33E-01	1.94E-02	SYBR Green I	None	3.25	6.53
D6	CK8	ABC2	22.03	21.86	0.17	Target	D4	1.05E-01	1.54E-02	SYBR Green I	None	3.37	6.57
D7	CK9	ABC2	21.29	21.74	0.45	Target	D7	1.84E-01	2.44E-02	SYBR Green I	None	3.11	6.49
D8	CK9	ABC2	21.75	21.74	0.45	Target	D7	1.42E-01	1.88E-02	SYBR Green I	None	3.45	5.36
D9	CK9	ABC2	22.18	21.74	0.45	Target	D7	9.94E-02	1.32E-02	SYBR Green I	None	3.34	6.57
D10	CK10	ABC2	21.51	21.29	0.21	Target	D10	1.99E-01	5.78E-02	SYBR Green I	None	3.66	6.8
D11	CK10	ABC2	21.09	21.29	0.21	Target	D10	2.66E-01	7.74E-02	SYBR Green I	None	3.93	6.96
D12	CK10	ABC2	21.26	21.29	0.21	Target	D10	2.37E-01	6.88E-02	SYBR Green I	None	3.17	6.52
E1	CK11	ACT1	18.86	18.91	0.05	Reference	E1	-	-	SYBR Green I	None	3.23	6.97
E2	CK11	ACT1	18.92	18.91	0.05	Reference	E1	-	-	SYBR Green I	None	2.54	6.56
E3	CK11	ACT1	18.96	18.91	0.05	Reference	E1	-	-	SYBR Green I	None	2.93	6.81
E4	CK12	ACT1	15.93	15.83	0.11	Reference	E4	-	-	SYBR Green I	None	2.93	6.81
E5	CK12	ACT1	15.84	15.83	0.11	Reference	E4	-	-	SYBR Green I	None	3.04	6.9
E6	CK12	ACT1	15.72	15.83	0.11	Reference	E4	-	-	SYBR Green I	None	3.04	6.9
E7	CK13	ACT1	18.81	18.62	0.2	Reference	E7	-	-	SYBR Green I	None	2.64	6.86
E8	CK13	ACT1	18.41	18.62	0.2	Reference	E7	-	-	SYBR Green I	None	2.9	6.8
E9	CK13	ACT1	18.65	18.62	0.2	Reference	E7	-	-	SYBR Green I	None	2.84	6.93
E10	CK14	ACT1	17.29	17.31	0.02	Reference	E10	-	-	SYBR Green I	None	2.89	6.95
E11	CK14	ACT1	17.32	17.31	0.02	Reference	E10	-	-	SYBR Green I	None	2.84	6.93
E12	CK14	ACT1	17.31	17.31	0.02	Reference	E10	-	-	SYBR Green I	None	2.74	6.88
F1	CK11	ABC2	21.68	21.82	0.16	Target	F1	1.47E-01	4.88E-02	SYBR Green I	None	3.4	6.56
F2	CK11	ABC2	21.78	21.82	0.16	Target	F1	1.37E-01	4.56E-02	SYBR Green I	None	3.23	6.46
F3	CK11	ABC2	22	21.82	0.16	Target	F1	1.17E-01	3.91E-02	SYBR Green I	None	3.2	6.42
F4	CK12	ABC2	18.79	18.78	0.11	Target	F4	1.39E-01	2.58E-02	SYBR Green I	None	3.04	6.51
F5	CK12	ABC2	18.67	18.78	0.11	Target	F4	1.20E-01	4.19E-02	SYBR Green I	None	2.28	6.01
F6	CK12	ABC2	18.88	18.78	0.11	Target	F4	1.30E-01	3.62E-02	SYBR Green I	None	3.47	6.75
F7	CK13	ABC2	21.83	22.28	0.44	Target	F7	1.08E-01	1.51E-02	SYBR Green I	None	3.14	6.51
F8	CK13	ABC2	22.71	22.28	0.44	Target	F7	5.89E-02	8.21E-03	SYBR Green I	None	3.44	6.78
F9	CK13	ABC2	22.3	22.28	0.44	Target	F7	7.82E-02	1.09E-02	SYBR Green I	None	3.46	6.74
F10	CK14	ABC2	22.55	22.46	0.08	Target	F10	2.63E-02	2.73E-03	SYBR Green I	None	3.31	6.45
F11	CK14	ABC2	22.44	22.46	0.08	Target	F10	2.84E-02	3.05E-03	SYBR Green I	None	3.05	6.3
F12	CK14	ABC2	22.4	22.46	0.08	Target	F10	2.92E-02	3.29E-03	SYBR Green I	None	3.3	6.4
G1	CK15	ACT1	19.35	19.29	0.16	Reference	G1	-	-	SYBR Green I	None	2.85	6.93
G2	CK15	ACT1	19.41	19.29	0.16	Reference	G1	-	-	SYBR Green I	None	3.02	6.91
G3	CK15	ACT1	19.1	19.29	0.16	Reference	G1	-	-	SYBR Green I	None	2.89	6.73
G4	CK16	ACT1	17.78	18.06	0.25	Reference	G4	-	-	SYBR Green I	None	3.06	6.84
G5	CK16	ACT1	18.21	18.06	0.25	Reference	G4	-	-	SYBR Green I	None	2.74	6.77
G6	CK16	ACT1	18.2	18.06	0.25	Reference	G4	-	-	SYBR Green I	None	2.7	6.71
G7	CK17	ACT1	18.15	18.25	0.1	Reference	G7	-	-	SYBR Green I	None	3.12	6.91
G8	CK17	ACT1	18.27	18.25	0.1	Reference	G7	-	-	SYBR Green I	None	3.14	6.96
G9	CK17	ACT1	18.34	18.25	0.1	Reference	G7	-	-	SYBR Green I	None	3.16	6.87
G10	CK18	ACT1	18.04	18.3	0.25	Reference	G10	-	-	SYBR Green I	None	2.57	6.67
G11	CK18	ACT1	18.34	18.3	0.25	Reference	G10	-	-	SYBR Green I	None	3.08	6.58
G12	CK18	ACT1	18.53	18.3	0.25	Reference	G10	-	-	SYBR Green I	None	2.92	6.94
H1	CK15	ABC2	23.85	23.91	0.18	Target	H1	4.23E-02	4.82E-03	SYBR Green I	None	3.42	6.54
H2	CK15	ABC2	23.77	23.91	0.18	Target	H1	4.47E-02	5.10E-03	SYBR Green I	None	3.44	6.58
H3	CK15	ABC2	24.11	23.91	0.18	Target	H1	3.53E-02	4.03E-03	SYBR Green I	None	2.98	6.28
H4	CK16	ABC2	27.95	25.64	2.32	Target	H4	1.06E-03	1.80E-04	SYBR Green I	None	3.77	6.87
H5	CK16	ABC2	25.65	25.64	2.32	Target	H4	1.37E-02	2.45E-03	SYBR Green I	None	3.7	6.31
H6	CK16	ABC2	23.32	25.64	2.32	Target	H4	2.62E-02	4.45E-03	SYBR Green I	None	3.23	6.47
H7	CK17	ABC2	22.4	22.48	0.08	Target	H7	5.61E-02	6.25E-03	SYBR Green I	None	3.55	6.69
H8	CK17	ABC2	22.49	22.48	0.08	Target	H7	5.02E-02	5.45E-03	SYBR Green I	None	3.72	6.68
H9	CK17	ABC2	22.56	22.48	0.08	Target	H7	5.33E-02	5.78E-03	SYBR Green I	None	3.53	6.65
H10	CK18	ABC2	23.5	23.37	0.18	Target	H10	2.69E-02	3.12E-03	SYBR Green I	None	3.15	6.41
H11	CK18	ABC2	23.44	23.37	0.18	Target	H10	2.81E-02	3.28E-03	SYBR Green I	None	3.39	6.58
H12	CK18	ABC2	23.17	23.37	0.18	Target	H10	3.38E-02	3.54E-03	SYBR Green I	None	3.45	6.66
