# Supplementary material for: Overexpression of Both ERG11 and ABC2 Genes Might Be Responsible for Itraconazole Resistance in Clinical Isolates of Candida krusei
Source: PLoS One. 2015 Aug 26;10(8):e0136185. doi: 10.1371/journal.pone.0136185 (PMC4550294; doi:10.1371/journal.pone.0136185)
Supplement: S3 Table — Ratio: the target gene ERG11 mRNA levels relative to the reference gene ACT1. (PDF) [file pone.0136185.s003.pdf]

Position	Sample Name	Gene Name	Cq	Cq Mean	Cq Error	Gene Type	Replicate Group	Ratio	Ratio Error	Dye	Failure	Slope	EPF
A1	CK1	ACT1	18.55	18.56	0.01	Reference	A1	-	-	SYBR Green I	None	2.47	6.83
A2	CK1	ACT1	18.57	18.56	0.01	Reference	A1	-	-	SYBR Green I	None	2.83	6.55
A3	CK1	ACT1	18.57	18.56	0.01	Reference	A1	-	-	SYBR Green I	None	2.17	6.18
A4	CK2	ACT1	18.75	18.65	0.16	Reference	A4	-	-	SYBR Green I	None	2.99	6.96
A5	CK2	ACT1	18.73	18.65	0.16	Reference	A4	-	-	SYBR Green I	None	3.17	6.83
A6	CK2	ACT1	18.46	18.65	0.16	Reference	A4	-	-	SYBR Green I	None	3.02	6.75
A7	CK4	ACT1	18.66	18.9	0.21	Reference	A7	-	-	SYBR Green I	None	2.98	6.99
A8	CK4	ACT1	19.01	18.9	0.21	Reference	A7	-	-	SYBR Green I	None	3.08	6.82
A9	CK4	ACT1	19.02	18.9	0.21	Reference	A7	-	-	SYBR Green I	None	2.87	6.61
A10	CK5	ACT1	15.94	15.97	0.06	Reference	A10	-	-	SYBR Green I	None	2.9	6.87
A11	CK5	ACT1	15.93	15.97	0.06	Reference	A10	-	-	SYBR Green I	None	2.8	6.49
A12	CK5	ACT1	16.03	15.97	0.06	Reference	A10	-	-	SYBR Green I	None	2.67	6.45
B1	CK1	ERG11	22.95	22.94	0.14	Target	B1	4.36E-02	5.34E-03	SYBR Green I	None	2.66	6.03
B2	CK1	ERG11	23.08	22.94	0.14	Target	B1	5.29E-02	5.87E-03	SYBR Green I	None	2.79	6.14
B3	CK1	ERG11	22.8	22.94	0.14	Target	B1	4.85E-02	5.13E-03	SYBR Green I	None	2.65	6.03
B4	CK2	ERG11	22.85	22.95	0.1	Target	B4	5.43E-02	6.09E-03	SYBR Green I	None	2.89	6.22
B5	CK2	ERG11	22.96	22.95	0.1	Target	B4	5.03E-02	5.65E-03	SYBR Green I	None	2.96	6.25
B6	CK2	ERG11	23.05	22.95	0.1	Target	B4	4.73E-02	5.31E-03	SYBR Green I	None	2.81	6.34
B7	CK4	ERG11	23.38	23.36	0.36	Target	B7	4.70E-02	5.28E-03	SYBR Green I	None	2.89	6.22
B8	CK4	ERG11	23	23.36	0.36	Target	B7	5.82E-02	8.27E-03	SYBR Green I	None	2.94	6.31
B9	CK4	ERG11	23.71	23.36	0.36	Target	B7	3.56E-02	5.05E-03	SYBR Green I	None	3.04	6.35
B10	CK5	ERG11	20.4	20.23	0.25	Target	B10	4.63E-02	1.77E-03	SYBR Green I	None	2.95	6.38
B11	CK5	ERG11	20.35	20.23	0.25	Target	B10	4.79E-02	1.83E-03	SYBR Green I	None	2.85	6.3
B12	CK5	ERG11	19.95	20.23	0.25	Target	B10	6.32E-02	2.41E-03	SYBR Green I	None	2.6	6.07
C1	CK6	ACT1	17.67	17.85	0.25	Reference	C1	-	-	SYBR Green I	None	2.82	6.81
C2	CK6	ACT1	17.74	17.85	0.25	Reference	C1	-	-	SYBR Green I	None	2.72	6.66
C3	CK6	ACT1	18.13	17.85	0.25	Reference	C1	-	-	SYBR Green I	None	2.46	6.83
C4	CK8	ACT1	18.72	18.85	0.12	Reference	C4	-	-	SYBR Green I	None	2.5	6.7
C5	CK8	ACT1	18.95	18.85	0.12	Reference	C4	-	-	SYBR Green I	None	2.58	6.91
C6	CK8	ACT1	18.88	18.85	0.12	Reference	C4	-	-	SYBR Green I	None	2.76	7.01
C7	CK9	ACT1	19.25	19.23	0.27	Reference	C7	-	-	SYBR Green I	None	2.83	6.95
C8	CK9	ACT1	18.96	19.23	0.27	Reference	C7	-	-	SYBR Green I	None	3.02	6.69
C9	CK9	ACT1	19.49	19.23	0.27	Reference	C7	-	-	SYBR Green I	None	2.96	7.01
C10	CK10	ACT1	-	-	-	Reference	C10	-	-	SYBR Green I	Failure	0.12	0.52
C11	CK10	ACT1	18.63	18.97	0.48	Reference	C10	-	-	SYBR Green I	None	3.06	6.6
C12	CK10	ACT1	19.31	18.97	0.48	Reference	C10	-	-	SYBR Green I	None	3.07	7.05
D1	CK6	ERG11	21.63	21.5	0.2	Target	D1	7.26E-02	1.25E-02	SYBR Green I	None	2.9	6.3
D2	CK6	ERG11	21.59	21.5	0.2	Target	D1	7.47E-02	1.28E-02	SYBR Green I	None	2.81	6.21
D3	CK6	ERG11	21.27	21.5	0.2	Target	D1	9.32E-02	1.60E-02	SYBR Green I	None	2.64	6.07
D4	CK8	ERG11	22.39	22.24	0.14	Target	D4	8.60E-02	7.03E-03	SYBR Green I	None	2.77	6.26
D5	CK8	ERG11	22.12	22.24	0.14	Target	D4	1.04E-01	8.47E-03	SYBR Green I	None	2.73	6.13
D6	CK8	ERG11	22.22	22.24	0.14	Target	D4	9.67E-02	7.90E-03	SYBR Green I	None	2.78	6.2
D7	CK9	ERG11	22.41	22.51	0.1	Target	D7	1.11E-01	2.03E-02	SYBR Green I	None	2.81	6.19
D8	CK9	ERG11	22.51	22.51	0.1	Target	D7	1.03E-01	1.90E-02	SYBR Green I	None	2.76	6.15
D9	CK9	ERG11	22.6	22.51	0.1	Target	D7	9.70E-02	1.78E-02	SYBR Green I	None	2.84	6.23
D10	CK10	ERG11	21.63	21.57	0.06	Target	D10	1.58E-01	2.45E-02	SYBR Green I	None	3.03	6.34
D11	CK10	ERG11	21.53	21.57	0.06	Target	D10	1.70E-01	2.98E-02	SYBR Green I	None	2.83	6.19
D12	CK10	ERG11	21.54	21.57	0.06	Target	D10	1.68E-01	2.37E-02	SYBR Green I	None	2.95	6.26
E1	CK11	ACT1	18.57	19.12	0.51	Reference	E1	-	-	SYBR Green I	None	2.88	6.89
E2	CK11	ACT1	19.59	19.12	0.51	Reference	E1	-	-	SYBR Green I	None	2.73	7.01
E3	CK11	ACT1	19.2	19.12	0.51	Reference	E1	-	-	SYBR Green I	None	2.44	6.81
E4	CK12	ACT1	18.42	18.59	0.18	Reference	E4	-	-	SYBR Green I	None	2.5	6.65
E5	CK12	ACT1	18.57	18.59	0.18	Reference	E4	-	-	SYBR Green I	None	2.75	6.7
E6	CK12	ACT1	18.77	18.59	0.18	Reference	E4	-	-	SYBR Green I	None	2.82	6.92
E7	CK13	ACT1	16.84	17.56	0.72	Reference	E7	-	-	SYBR Green I	None	2.67	6.52
E8	CK13	ACT1	18.27	17.56	0.72	Reference	E7	-	-	SYBR Green I	None	2.67	6.94
E9	CK13	ACT1	17.57	17.56	0.72	Reference	E7	-	-	SYBR Green I	None	2.84	6.82
E10	CK14	ACT1	17.13	17.25	0.12	Reference	E10	-	-	SYBR Green I	None	3.11	6.11
E11	CK14	ACT1	17.37	17.25	0.12	Reference	E10	-	-	SYBR Green I	None	2.92	5.68
E12	CK14	ACT1	17.25	17.25	0.12	Reference	E10	-	-	SYBR Green I	None	2.63	6.79
F1	CK11	ERG11	21.94	21.84	0.12	Target	F1	1.42E-01	5.05E-02	SYBR Green I	None	2.85	6.26
F2	CK11	ERG11	21.71	21.84	0.12	Target	F1	1.66E-01	5.93E-02	SYBR Green I	None	2.81	6.25
F3	CK11	ERG11	21.86	21.84	0.12	Target	F1	1.50E-01	5.34E-02	SYBR Green I	None	2.67	6.09
F4	CK12	ERG11	22.33	22.11	0.2	Target	F4	7.47E-02	9.09E-03	SYBR Green I	None	2.63	6.04
F5	CK12	ERG11	21.94	22.11	0.2	Target	F4	9.79E-02	1.19E-02	SYBR Green I	None	2.73	6.14
F6	CK12	ERG11	22.06	22.11	0.2	Target	F4	9.00E-02	1.10E-02	SYBR Green I	None	2.9	6.26
F7	CK13	ERG11	22.01	22.13	0.13	Target	F7	4.58E-02	2.27E-02	SYBR Green I	None	2.77	6.2
F8	CK13	ERG11	22.26	22.13	0.13	Target	F7	3.85E-02	1.91E-02	SYBR Green I	None	2.69	6.07
F9	CK13	ERG11	22.13	22.13	0.13	Target	F7	4.21E-02	2.09E-02	SYBR Green I	None	2.7	6.1
F10	CK14	ERG11	23.58	23.61	0.03	Target	F10	1.24E-02	1.45E-03	SYBR Green I	None	2.78	6.13
F11	CK14	ERG11	23.63	23.61	0.03	Target	F10	1.20E-02	1.46E-03	SYBR Green I	None	2.79	6.15
F12	CK14	ERG11	23.62	23.61	0.03	Target	F10	1.23E-02	1.45E-03	SYBR Green I	None	2.78	6.11
G1	CK15	ACT1	18.82	19.05	0.2	Reference	G1	-	-	SYBR Green I	None	2.98	6.67
G2	CK15	ACT1	19.12	19.05	0.2	Reference	G1	-	-	SYBR Green I	None	2.96	6.67
G3	CK15	ACT1	19.2	19.05	0.2	Reference	G1	-	-	SYBR Green I	None	2.95	6.65
G4	CK16	ACT1	16.44	16.43	0.31	Reference	G4	-	-	SYBR Green I	None	2.77	6.88
G5	CK16	ACT1	16.11	16.43	0.31	Reference	G4	-	-	SYBR Green I	None	2.87	6.61
G6	CK16	ACT1	16.73	16.43	0.31	Reference	G4	-	-	SYBR Green I	None	2.74	6.89
G7	CK17	ACT1	16.27	16.52	0.26	Reference	G7	-	-	SYBR Green I	None	3.01	7.07
G8	CK17	ACT1	16.5	16.52	0.26	Reference	G7	-	-	SYBR Green I	None	2.9	6.79
G9	CK17	ACT1	16.78	16.52	0.26	Reference	G7	-	-	SYBR Green I	None	2.67	6.97
G10	CK18	ACT1	18.68	18.58	0.26	Reference	G10	-	-	SYBR Green I	None	2.94	7.04
G11	CK18	ACT1	18.28	18.58	0.26	Reference	G10	-	-	SYBR Green I	None	3.05	6.64
G12	CK18	ACT1	18.77	18.58	0.26	Reference	G10	-	-	SYBR Green I	None	3.18	6.93
H1	CK15	ERG11	24.52	24.51	0.08	Target	H1	2.29E-02	3.64E-03	SYBR Green I	None	2.95	6.23
H2	CK15	ERG11	24.43	24.51	0.08	Target	H1	2.40E-02	3.33E-03	SYBR Green I	None	2.82	6.15
H3	CK15	ERG11	24.58	24.51	0.08	Target	H1	2.16E-02	3.00E-03	SYBR Green I	None	2.72	6.11
H4	CK16	ERG11	22.14	22.02	0.1	Target	H4	1.91E-02	4.10E-03	SYBR Green I	None	2.61	6.06
H5	CK16	ERG11	21.94	22.02	0.1	Target	H4	2.19E-02	4.71E-03	SYBR Green I	None	2.85	6.24
H6	CK16	ERG11	21.99	22.02	0.1	Target	H4	2.12E-02	4.55E-03	SYBR Green I	None	2.82	6.21
H7	CK17	ERG11	21.22	21.49	0.27	Target	H7	3.84E-02	6.80E-03	SYBR Green I	None	2.92	6.29
H8	CK17	ERG11	21.75	21.49	0.27	Target	H7	2.66E-02	4.71E-03	SYBR Green I	None	2.93	6.27
H9	CK17	ERG11	21.51	21.49	0.27	Target	H7	3.26E-02	5.43E-03	SYBR Green I	None	3.1	6.27
H10	CK18	ERG11	23.79	23.62	0.22	Target	H10	2.70E-02	4.87E-03	SYBR Green I	None	3.05	6.34
H11	CK18	ERG11	23.71	23.62	0.22	Target	H10	2.85E-02	5.15E-03	SYBR Green I	None	3.1	6.38
H12	CK18	ERG11	23.37	23.62	0.22	Target	H10	3.61E-02	6.52E-03	SYBR Green I	None	3.12	6.39
